# Leptin Receptor Expressing Neurons in the Substantia Nigra Regulate Locomotion, and in The Ventral Tegmental Area Motivation and Feeding

**DOI:** 10.3389/fendo.2021.680494

**Published:** 2021-07-01

**Authors:** Véronne A. J. de Vrind, Lisanne J. van ‘t Sant, Annemieke Rozeboom, Mieneke C. M. Luijendijk-Berg, Azar Omrani, Roger A. H. Adan

**Affiliations:** ^1^ Brain Center Rudolf Magnus, Department of Translational Neuroscience, University Medical Center Utrecht and University Utrecht, Utrecht, Netherlands; ^2^ Institute of Neuroscience and Physiology, The Sahlgrenska Academy at the University of Gothenburg, Gothenburg, Sweden

**Keywords:** leptin, dopamine, chemogenetics, midbrain, substantia nigra, ventral tegmental area

## Abstract

Leptin is an anorexigenic hormone, important in the regulation of body weight. Leptin plays a role in food reward, feeding, locomotion and anxiety. Leptin receptors (LepR) are expressed in many brain areas, including the midbrain. In most studies that target the midbrain, either all LepR neurons of the midbrain or those of the ventral tegmental area (VTA) were targeted, but the role of substantia nigra (SN) LepR neurons has not been investigated. These studies have reported contradicting results regarding motivational behavior for food reward, feeding and locomotion. Since not all midbrain LepR mediated behaviors can be explained by LepR neurons in the VTA alone, we hypothesized that SN LepR neurons may provide further insight. We first characterized SN LepR and VTA LepR expression, which revealed LepR expression mainly on DA neurons. To further understand the role of midbrain LepR neurons in body weight regulation, we chemogenetically activated VTA LepR or SN LepR neurons in LepR-cre mice and tested for motivational behavior, feeding and locomotion. Activation of VTA LepR neurons in food restricted mice decreased motivation for food reward (p=0.032) and food intake (p=0.020), but not locomotion. In contrast, activation of SN LepR neurons in food restricted mice decreased locomotion (p=0.025), but not motivation for food reward or food intake. Our results provide evidence that VTA LepR and SN LepR neurons serve different functions, i.e. activation of VTA LepR neurons modulated motivation for food reward and feeding, while SN LepR neurons modulated locomotor activity.

## Introduction

The current obesity epidemic is a result of overconsumption that exceeds energy requirements. With no truly effective treatments, there is a need for further mechanistic insights into body weight regulation. Hedonic feeding is characterized by the increased consumption of palatable foods and implicates increased motivation for these foods, which ultimately results in obesity (1). Food deprivation or dieting is known to increase motivation for food, which counteracts weight loss (2). In light of the obesity epidemic, it is of interest to gain further knowledge on mechanisms underlying increased motivation for (palatable) food.

Leptin is an anorexic adipose-tissue derived hormone with a central role in body weight regulation. Leptin is secreted in levels proportional to the amount of adipose tissue, i.e. fasting decreases leptin levels, while weight gain increases leptin levels (3–5). Leptin is known to regulate body weight by reducing feeding and increasing energy expenditure (6). Accumulating evidence shows that leptin is also involved in reducing motivation for food in both humans and rodents (7–11). The midbrain, comprising of the ventral tegmental area (VTA) and substantia nigra (SN), is associated with motivational behavior and expresses the leptin receptor (LepR), but it is unclear whether and which of these regions mediate leptin’s effect on motivational behavior for food reward (12–14).

Since VTA DA neurons are important for motivational behaviour, VTA DA neurons expressing LepR were proposed to mediate the effect of leptin on food reward (15–18). However, there are data that challenge this idea. An important reward associated pathway are VTA DA neurons projecting to the nucleus accumbens (NAc), but only few VTA-LepR neurons project to the NAc (19). Instead, VTA LepR neurons primarily project to the central amygdala, and these neurons mediate effects of leptin on central amygdala associated behaviors, such as anxiety (18). Indeed, leptin infusion into the VTA of mice decreased anxiety-like behavior (20). Together, these data suggest that VTA (DA) LepR neurons modulate anxiety-like and not motivational behavior. Yet, knockdown of LepR in the whole midbrain increased motivational behavior as assessed by operant responding (21). This suggests that another population of midbrain LepR neurons modulate motivation. In the SN, LepR are almost exclusively expressed on SN DA neurons (18) and SN DA neurons have been implicated in motivational behavior (22). Optogenetic activation of either VTA or SN dopamine neurons demonstrated that dopamine neurons in the VTA and SN exhibited divergent conditioned motivational functions: VTA, but not SN dopamine neurons conferred a signal that made cues attractive and reinforcing; SN dopamine neurons conferred a more general movement invigoration signal: cues paired with their activation evoked vigorous movement not directed at the cue (23). Thus, SN dopamine neurons play a role in motivation and an involvement of SN LepR neurons in mediating the effect of leptin on motivational behavior is possible, but has not yet been determined.

Besides the role of midbrain LepR neurons in motivation for food reward, this region has also been implicated in other aspects of leptin’s behavioral effects such as feeding and energy expenditure. Leptin injection into the VTA decreased feeding (15, 24–26) and blocking or decreasing leptin signaling in the VTA increased feeding (15, 25). However, inhibiting leptin signaling in the whole midbrain (21) or specifically in DA neurons of the midbrain (27, 28) did not impact on feeding. These results suggest that perhaps effects on feeding are mediated by non-DA neurons in the VTA. In addition, it remains unknown whether SN LepR neurons contribute to the anorexic effect of leptin.

Effects of midbrain LepR neurons on locomotor activity are contradicting. Whereas leptin infusion into the VTA had no effect on locomotion in ad libitum fed rats (15), RNA interference knockdown of LepR in VTA LepR neurons or ablation of STAT3 in midbrain (SN and VTA) DA neurons increased locomotion (15, 27). One reason for a lack of effect of leptin infusion on locomotion is that endogenous leptin masked effects on locomotion in these experiments since intra-VTA leptin injections were given to ad libitum fed rats that already have high levels of leptin (15). Alternatively, SN LepR neurons could mediate effects of leptin on locomotion. Thus, it remains unclear which midbrain LepR population modulates locomotion.

The aim of the current study was to further insight into the mechanisms underlying the effect of leptin in the midbrain and its involvement in body weight regulation. We started with characterizing the expression of LepR in the midbrain. Next, two questions were of particular interest for behavioral experiments: 1) whether SN LepR neurons modulate motivational, locomotor and feeding behavior and 2) whether VTA LepR neurons mediate anxiety-like, feeding and locomotor behavior. To address these questions, we selectively activated VTA or SN neurons that express leptin receptor, by injecting a viral vector that expresses the CNO receptor hM3Dq (also referred to as chemogenetics) in either the VTA or SN of LepR cre mice. We directly compared behavioral effects of chemogenetically activating VTA LepR or SN LepR neurons on motivational behavior for food reward, feeding, locomotion and anxiety in LepR-cre mice.

## Materials and Methods

All experiments were approved by the Animal Experimentation Committee of the University Utrecht and were carried out in agreement with Dutch Law (Herziene Wet op Dierproeven, Art 10.a2, 2014) and European regulations (Guideline 2010/63/EU).

### Subjects

**Figure 1:**
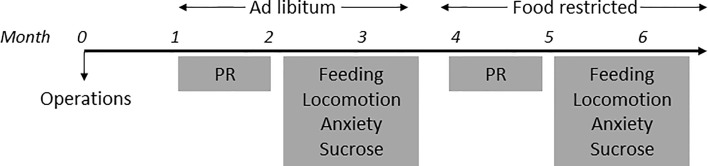
Timeline of experiments.

In house bred, adult male homozygote LepRb-Cre transgenic mice (stock #008320, B6.129-Leprtm2(cre)Rck/J, The Jackson Laboratory, USA) were used for behavioral experiments. Mice were housed individually in plastic cages (Type II L, 365x207x140mm, 530cm^2^, Tecniplast, Italy) in a temperature (21 ± 2°C) and humidity (60-70%) controlled room on a reversed day/night (9:00 AM light OFF, 9:00 PM light ON) schedule. We tested all mice first in ad libitum situations (**Figure 1**) and then under food restriction, because leptin levels are correlated with the amount of fat tissue in mice (3, 5) and leptin influences midbrain DA activity (15, 16, 29). Ad libitum fed mice were given ad libitum access to chow (3,1Kcal/gr, Standard Diet Service, UK) and water. Food-restricted mice were given chow (~3 pieces of standard rodent chow of ±1.3 grams each) after behavioral tasks were performed and maintained on ~90% of the original body weight by adapting the amount of chow given per day. For analysis of DA (determined by staining for TH immunoreactivity) and LepR co-localization, LepRb-Cre mice were crossed with a mouse line that expresses robust tdTomato fluorescence following Cre-mediated recombination (stock # 007914, B6.Cg-Gt(ROSA)26Sortm14(CAG-tdTomato)Hze/J, The Jackson Laboratory, USA). Male mice homozygote for the LepRb-Cre allele and heterozygote for the TdTom allele (“LepR/tdTomato” mice, n=3) were socially housed on a normal day/night cycle (7:00AM lights ON) with ad libitum access to chow and water.

### Surgical Procedures

At least 30min prior to anesthetization, mice were given carprofen (5mg/kg, subcutaneous (s.c.), Carporal, AST Farma BV). Mice were anesthetized with an intraperitoneal (i.p.) injection of ketamine (75mg/kg, Narketan, Vetoquinol BV) and medetomidine (1mg/kg, SedaStart, AST Farma BV). Mice were given eye cream (CAF, CEVA Sante Animale BW) and placed in a stereotactic frame (Kopf Instruments, USA). An incision was made in the skin along the midline of the skull and additional analgesia was applied by spraying Xylocaine (lidocaine 100mg/ml, AstraZeneca BV) on the skull. Microinjections of AAV5-hSyn-DIO-hM3DGq-mCherry (0.3ul/side, 3.0*10^9^vp/ul, UNC Vector Core, USA) or AAV-Ef1a-DIO-hChR2-eYFP (“control” mice, 0.3ul/side, 3.0*10^9vp/ul, UNC Vector Core, USA, n=6) were performed bilaterally in the substantia nigra (-3.3 AP, +1.5 ML, -4.4 DV, 0° angle, n=6) or the ventral tegmental area (-3.2 AP, +1.5 ML, -4.8 DV, 10° angle, n=6) at a rate of 0.1ul/min per side followed by a 10min period before retracting the needles. For assessment of SN LepR projection sites mice were injected with AAV-Ef1a-DIO-ChR2-eYFP (0.3ul/side, 3.0*10^9^vp/ul, UNC Vector Core, USA) in the SN (n=4). After the operation, mice were given atipamezole (2.5mg/kg, i.p., SedaStop, AST Farma BV) and saline for rehydration. The following 2 days, mice were given carprofen (5mg/kg, s.c.) and were allowed to recover for at least 1 week after operations. To ensure viral expression, testing commenced 3 weeks after operations were performed. Thus, the mice were exposed to behavioral testing at least four weeks after surgery.

### Drugs

Clozapine N-oxide (CNO 99%, AK Scientific, Inc., USA) was dissolved in 0.9% saline and injected i.p. with a commonly used dose of 1.0mg/kg (and 3.0mg/kg for operant testing). For all experiments, each mouse received saline and CNO injections in a latin-square design. Mice were injected with saline or CNO 30 minutes prior to the start of a behavioral task.

### Behavioral Procedures

All mice were tested in every behavioral experiment. On test days only one single behavioral experiment was performed (**Figure 1**). Furthermore, between two test days on which mice were injected, there was at least one day on which mice were not injected.

#### Progressive Ratio Operant Behavior

Mouse operant boxes (ENV-307W, Med Associates Inc., USA) fitted with 2 levers, a cue light above the active lever (AL), a house light, a speaker and a liquid receptacle were used. Throughout all sessions, when the number of AL presses to complete a ratio was reached, the house light and a tone were presented for 5sec, after which a sucrose reward was delivered for 2sec (38ul, 20% w/v sugar solution in tap water). During the training phase, mice were food restricted to ~90% of their original body weight. The first day, mice were habituated for 15min to an operant box, in which we placed a droplet of sugar solution (20% w/v) in the receptacle. The next day, operant training started with a fixed ratio (FR) 1 paradigm for 30min/session. Once mice learned to press on the active lever >20x and <10% on the inactive lever, mice were switched to FR3 (30min/session) and then FR5 (60min/session). Then, once >60 rewards were earned and <10% of the presses were made on the inactive lever, training was switched to the progressive ratio (PR, 60min/session) and mice were returned to ad libitum feeding. PR schedule was based on the formula: 5*e^(x*0.2)^-5, rounded to the nearest integer, where x is the position in the ratio sequence (30). Testing with saline/CNO started when PR performance appeared stable, i.e. over 3 days of training no more than ±1 reward from average and no incremental increase or decrease. Operant training and testing were performed during the first 3 hours of the dark phase. Injections were given 30min prior to the start of the PR task. With this test we measured the amount of active lever presses made during 60min of the PR task.

#### Free Access 20% Sucrose Consumption

Mice were trained to lick for 20% (w/v) granulated sugar solution in an operant box fitted with a spout connected to a pump which delivered 8ul 20% sugar solution upon every detected lick. Mice were trained 4 times prior to testing. Training and testing was performed 3-5h into the dark phase and lasted 30min. On test days, mice were injected with CNO/saline 30min before the start of the test. With this test we measured the number of detected licks during 30 min testing.

#### Feeding

To simplify finding pieces of chow at measurements, all mice were habituated twice to a second mouse cage, in which no bedding, but only 3 tissues and a water bottle were present, hereafter referred to as ‘feeding cage’. During habituation we also habituated the mice to the experimenter taking chow out of the cage with the mice remaining in the cage. Mice were injected with saline/CNO 30min prior to the onset of the dark phase and were directly placed in the feeding cage with a pre-weighed amount of chow on the cage floor. Food intake was measured at 1, 2, 3, 5 and 7h post injection. At these timepoints, we weighed the pieces of chow with mice still in the feeding cage as they were used to from habituation to keep stress levels to a minimum. With this test we measured the amount of chow eaten in grams at the different timepoints.

#### Locomotion

All mice were habituated 2x1h to an individual plastic cage (Type III H, 425x266x185mm, 800cm^2^, Tecniplast, Italy) prior to testing on separate days. The cages were surrounded by white carton to prevent interaction between mice. On the test day, mice were injected with either saline/CNO and placed in the behavioral testing room. 30min later, mice were placed in their own locomotion cage and horizontal movement was tracked using a camera placed above the cages that was coupled to a computer running Ethovision 7 (Noldus Information Technology, the Netherlands). Locomotion tests commenced 4-5h into the dark phase and lasted 1h. With this test we measured the distance mice had travelled in meters in 1h.

#### Anxiety

Mice were injected with either saline/CNO 30min prior to testing and placed in the front room of the behavioral room where tests would be performed. Mice were lifted by their tails and placed in the center of the elevated plus maze in a brightly lit room and tracked using Ethovision (Noldus, Wageningen) for 5min. Anxiety tests were performed 6-7h into the dark phase. A previous study reported that longer intervals between anxiety tests led to reliable retesting of anxiety (31), so we separated 2 test days by at least 3 weeks. With this test we measured the amount of time mice spent in the open and dark arms and the amount of crossings made.

### Tissue Preparation and Immunohistochemical Analysis

Mice were anesthetized with Euthanimal (200mg/ml, Euthanimal, Alfasan BV, the Netherlands) and transcardially perfused with 1x phosphate-buffered saline (PBS) followed by 4% paraformaldehyde in 1xPBS (PFA). Brains were dissected and kept in 4%PFA for 24h at 4°C, after which they were transferred to 30% sucrose in 1xPBS for at least 48h at 4°C. Using a cryostat, the brains were then sectioned to 40um slices and stored in 1xPBS with 0.01% sodium azide. Slices were washed 3x10min in 1xPBS and then blocked for 1h in 1xPBS containing 10% normal goat serum and 0,25% Triton-X100. Slices were then placed in 1xPBS containing the primary antibodies (Rabbit anti-dsRed 1:500, #632496, Clontech, Takara Bio USA Inc, USA; Mouse anti-Th 1:500, MAB318, Milipore) and 2% normal goat serum overnight at 4°C. At room temperature, slices were washed 3x10min in 1xPBS and placed in 1xPBS containing the secondary antibodies (Goat anti-Rabbit 568, 1:500, #ab175471, Abcam plc, UK; Goat anti-Mouse 488 1:500, ab150113, Abcam plc, UK) and 2% normal goat serum for 2h. Finally, slices were washed in 1xPBS and mounted onto slides, dried and covered using Fluorsave (EMD Millipore Corporation, USA) and a coverslip. Images were collected on an epifluorescent microscope (Axio Scope A1, Zeiss, Germany). For behavioral mice, at least 3 representative images were analyzed per mouse from sections ranging from -2.54mm to -3.88mm (bregma).

### Data Analysis

Behavioral data was analyzed using Microsoft Excel and GraphPad Prism (version 7.05, GraphPad Software, Inc., USA). Paired t-tests and one- or two-way repeated measures ANOVA and Bonferroni’s multiple comparisons tests were used where applicable. A significance criterion of p<0.05, two-tailed, was adopted in all the statistical analyses.

## Results

### The Majority of Midbrain LepR Neurons Are Dopaminergic

**Figure 2:**
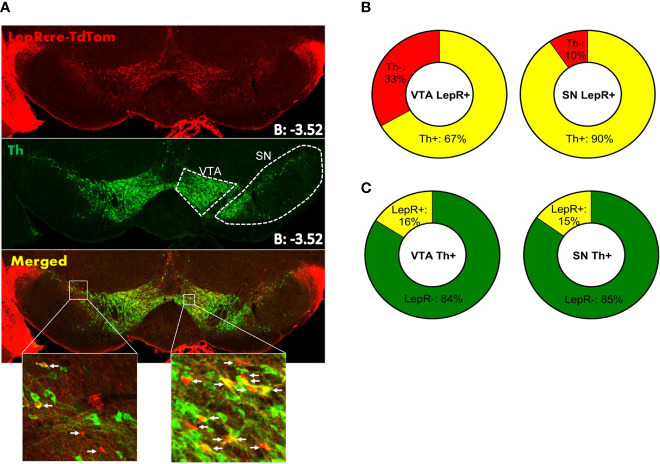
Immunohistochemistry on LepR-cre x TdTomato mice (n=3). **(A)** Both SN and VTA expressed LepR neurons (arrows in insets indicate LepR). **(B)** The majority of LepR neurons co-localized with TH (VTA: 67%, SN: 90%) **(C)** Of all TH neurons, only 16% in the VTA and 15% in the SN expressed the LepR.

To verify previous reports of co-localization of LepR on DA neurons, we assessed LepR expression in the midbrain. To identify LepR neurons in the midbrain, we crossed LepR-cre mice onto the ROSA26-tdTomato reporter mouse line, which allowed robust tdTomato fluorescence following Cre-mediated recombination. Tyrosine hydroxylase (TH) is the rate limiting enzyme in catecholamine production and considering that the VTA and SN are known to be DA producing structures, TH is used as marker for DA neurons. TH- and LepR-tdTomato immunoreactivity revealed that in the VTA 67 ± 1.0% and in the SN 89 ± 3.5% of LepR/tdTomato neurons co-localized with TH (**Figures 2A, B**). Of all TH neurons, only few co-localized with LepR/tdTomato in either region: 16 ± 1.3% in the VTA and 15% in the SN (n=3, **Figure 2C**). Thus, DA neurons that express LepR represented a minority of all DA neurons in VTA and SN, which is consistent with previous reports (15, 17, 18, 32). The majority of SN and VTA neurons expressing leptin receptor (LepR-tdTomato) are DA neurons.

**Figure 3:**
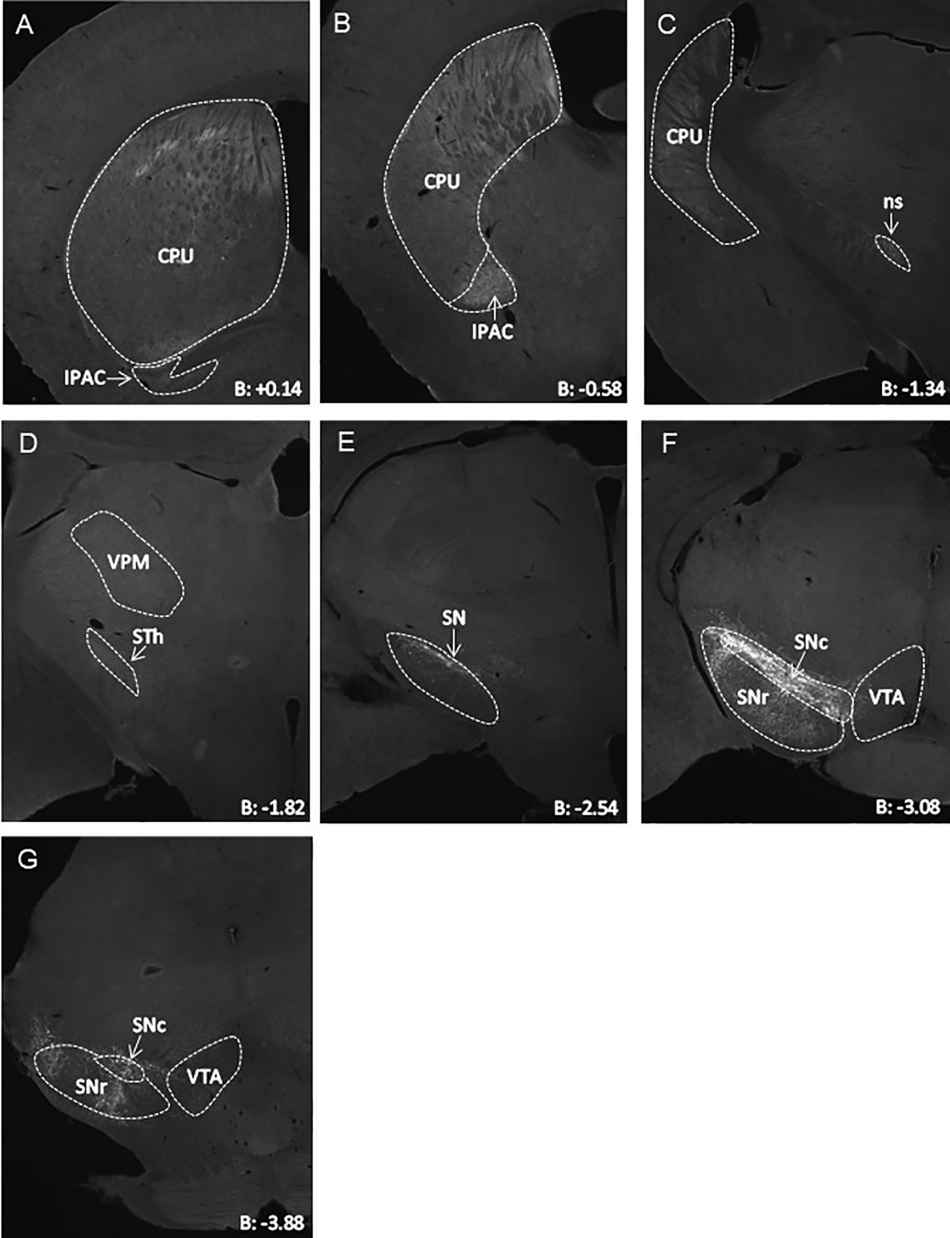
Representative AAV-DIO-ChR2-eYFP tracing of projections from SN LepR neurons in LepR-cre mice (n=4). **(A–G)** eYFP expression in CPU ++, IPAC ++, STh +, VPM + and VTA +/- to which SN LepR neurons sent detectable projections (++ strong eYFP, + eYFP present, +/- scarce eYFP). In **(E–G)**, eYFP soma and tracks within the SN. B = bregma; CPU, caudate putamen; IPAC, interstitial nucleus of the posterior limb of the anterior commissure; ns, nigrostriatal bundle; SN, substantia nigra; SNc, SN pars compacta; SNr, SN pars reticulate; STh, subthalamic nucleus; VPM, Ventral posteromedial thalamus; VTA, ventral tegmental area.

We next aimed to assess the projection areas of midbrain LepR neurons. It has been established that VTA LepR neurons primarily project to the central amygdala, while very few project to the NAc (18, 19). SN LepR neurons have been shown to project to the caudate putamen (18) and we sought to further extend on this knowledge. We identified projections of SN LepR neurons by injecting LepR-cre mice with AAV-DIO-ChR2-eYFP into the SN, which also labels axonal projections. We found that SN LepR neurons projected heavily to the caudate putamen and further observed projections to the interstitial nucleus of the posterior limb of the anterior commissure (IPAC) (**Figures 3A–C**). Some eYFP expression was found in the thalamus and in the VTA (**Figures 3D, F**). Finally, labeling of axons with eYFP was also observed within SN pars compacta, SN pars reticula and lateral SN (**Figures 3E–G**). We cannot exclude that the observed staining was due to fibers passing the areas. However the density of fibers in these areas was remarkable compared to surrounding areas.

**Figure 4:**
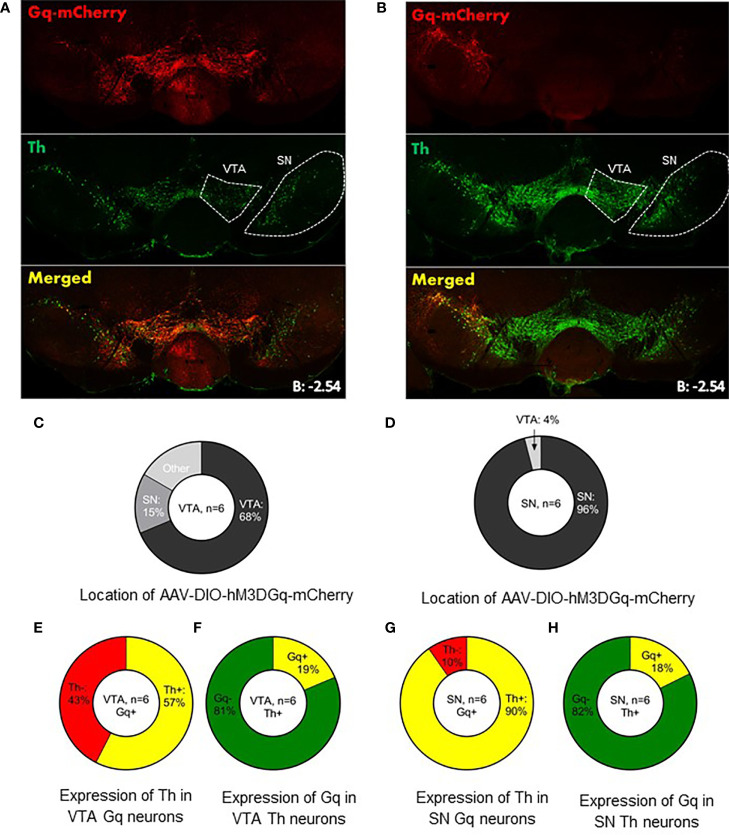
Expression of AAV-DIO-hM3DGq-mCherry in behavioral animals. Representative example of AAV-DIO-hM3DGq-mCherry expression in **(A)** VTA LepR-Gq mice (n=6) and **(B)** SN LepR-Gq mice (n=6). **(C, D)** Analysis of the location of Gq-mCherry expression showed that the majority of Gq-neurons were found in the targeted region. **(E)** Analysis of Gq and TH in VTA LepR-Gq mice showed that 57% of VTA Gq neurons co-localized with TH-immunoreactivity. **(F)** 19% of all VTA TH neurons co-localized with Gq. **(G)** 96% of SN Gq neurons co-localized with TH-immunoreactivity. **(H)** Of all SN Th neurons, 18% co-localized with Gq-mCherry.

For behavioral experiments we injected AAV-DIO-hM3DGq-mCherry in the VTA (n=6) or SN (n=6) of LepR-cre mice to generate VTA LepR-Gq and SN LepR-Gq mice. In earlier studies we already demonstrated that in slices of animals with expression of this DREADD receptor in midbrain DA neurons, CNO depolarized DA neurons (33). We examined whether expression of hM3DGq-mCherry was representative for SN LepR or VTA LepR neurons. hM3DGq-mCherry was found mainly in the targeted region, but due to viral spread, some expression was seen in surrounding regions which express LepR-cre: of all hM3DGq-mCherry expressing neurons 68 ± 1.1% were localized in the VTA of VTA LepR-Gq mice and 96 ± 1.1% were localized in the SN of SN LepR-Gq mice (**Figure 4**). The percentage of TH neurons expressing hM3DGq-mCherry or the percentage of hM3DGq-mCherry neurons expressing TH were similar to the numbers presented above of TH/LepR co-localization in LepR-tdTomato mice. Thus, hM3DGq-mCherry targeted neurons were representative for LepR neurons of the VTA or SN.

### Control Mice Do Not Show Behavioral Effects After CNO Injections Compared to Saline

Studies show that non-specific effects of CNO may occur in rats and mice due to reverse-metabolism to its parent compound clozapine (34). To ensure this is not the case in our study, we injected AAV-Ef1a-DIO-hChR2-eYFP into the VTA or SN of LepR-cre mice (n=6). This results in expression of channelrhodopsin, which does not respond to CNO or clozapine. As we expected, CNO injections in control mice had no effect on any behavioral parameter tested (**Supplemental Figure**, n=6). Therefore, we conclude that behavioral effects observed in Gq-injected mice are the result of enhanced neuronal activation in VTA LepR-Gq or SN LepR-Gq mice and not due to effects of reverse-metabolism of CNO to clozapine.

### Motivation for Sugar Reward Decreased Upon Activation of VTA LepR-Gq Neurons in Food Restricted Mice

**Figure 5:**
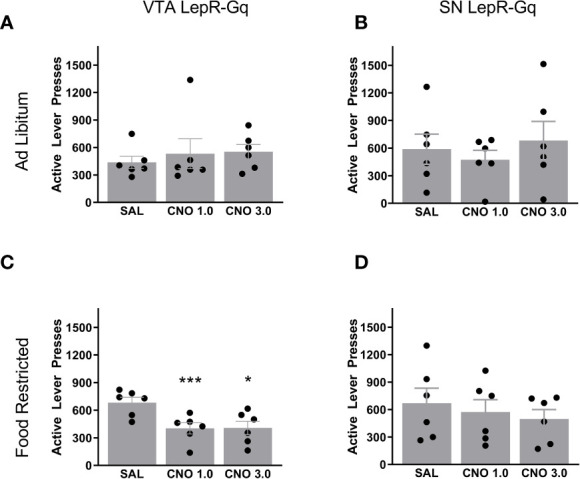
Active lever presses upon chemogenetic activation of VTA LepR or SN LepR neurons. In ad libitum fed mice, CNO (1.0 and 3.0mg/kg) did not affect active lever presses made in **(A)** VTA LepR-Gq (n=6) or **(B)** SN LepR-Gq mice (n=6). **(C)** Food restricted VTA LepR-Gq mice (n=6) decreased active lever presses after CNO compared to saline injections. **(D)** CNO did not affect active lever presses in food restricted SN LepR-Gq mice (n=6). Mean ± SEM. *p<0.05, ***p<0.001.

To test for motivation, we trained mice to press for a 20% sugar solution reward in the PR task. Once stable, mice were injected with saline and 2 doses of CNO (1.0 and 3.0mg/kg). Ad libitum fed VTA LepR-Gq mice did not alter operant responding after CNO injections compared to saline (**Figure 5A**), but when food restricted, VTA LepR-Gq mice decreased the amount of active lever presses made during the PR task (*F*(1.110,5.549)=7.920, p=0.032, n=6, **Figure 5C**). Further analyses revealed that both CNO doses significantly decreased performance compared to saline (CNO 1.0mg/kg, p=0.0007; CNO 3.0mg/kg, p=0.019). Operant behavior was not affected upon chemogenetic activation of SN LepR-Gq neurons (n=6, **Figures 5B, D**).

### Free Consumption of Sucrose Solution Was Not Affected by VTA LepR or SN LepR Activation

**Figure 6:**
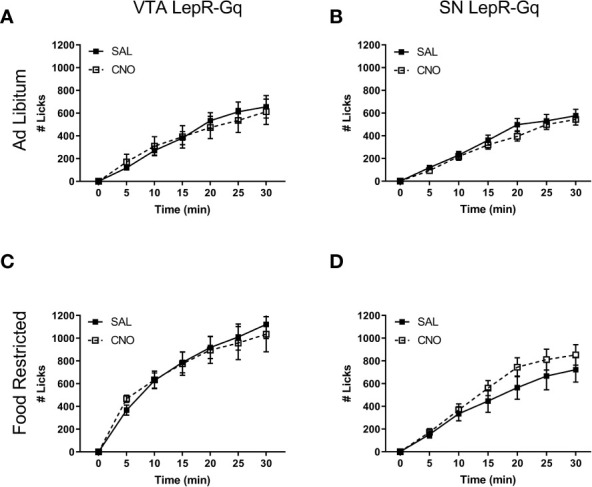
Free 20% sucrose solution consumption upon chemogenetic activation of VTA LepR or SN LepR neurons. CNO (1.0mg/kg) did not affect sucrose solution consumption in ad libitum fed **(A)** VTA LepR-Gq (n=6) and **(B)** SN LepR- Gq (n=6) or food restricted **(C)** VTA LepR-Gq (n=6) and **(D)** SN LepR-Gq (n=6). Mean ± SEM.

To dissociate motivational behavior from consumption, we assessed free consumption of a 20% sucrose solution. Free consumption was not affected by activation of either VTA LepR or SN LepR, indicating that decreased motivation was driven by motivational behavior without affecting consummatory behavior (**Figure 6**). The apparent difference in baseline sucrose consumption between mice injected in the VTA compared to the SN is most likely due to the fact that these were different batches of mice that were tested at different time points.

### Chow Consumption Decreased Upon Activation of VTA LepR-Gq Neurons in Food Restricted Mice

**Figure 7:**
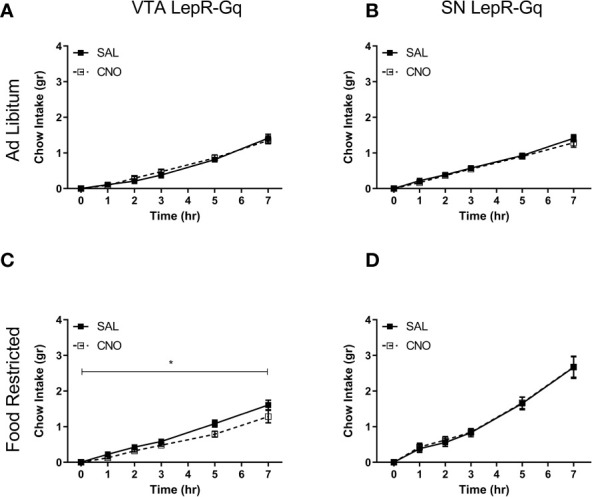
Consumption of chow upon chemogenetic activation of VTA LepR or SN LepR neurons. Chow intake was not affected by CNO (1.0mg/kg) injections in ad libitum fed **(A)** VTA LepR-Gq (n=6) or **(B)** SN LepR-Gq mice (n=6). **(C)** CNO decreased cumulative chow intake in food restricted VTA LepR-Gq mice (n=6). **(D)** CNO did not affect feeding in food restricted SN LepR-Gq mice (n=6). Mean ± SEM. *p<0.05.

We next tested whether midbrain LepR neurons modulated feeding by measuring chow intake over 7h. Ad libitum fed mice did not alter consumption of chow after chemogenetically activating VTA LepR-Gq (**Figure 7A**) or SN LepR-Gq (**Figure 7B**) neurons. In food restricted VTA LepR-Gq mice, CNO decreased cumulative chow intake compared to saline (main effect of Injection *F* (1,5)=9.138, p=0.029, **Figure 7C**), but food intake was unaffected in food restricted SN LepR-Gq mice (**Figure 7D**).

### Locomotion Decreased Upon Activation of SN LepR-Gq Neurons in Food Restricted Mice

**Figure 8:**
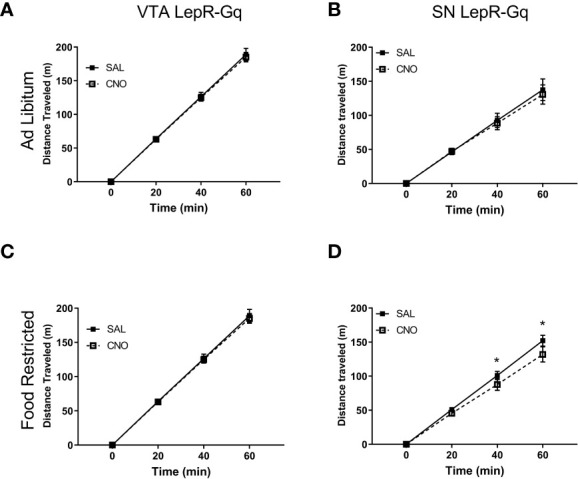
Locomotor activity upon chemogenetic activation of VTA LepR or SN LepR neurons. Locomotion was not affected by CNO (1.0mg/kg) injections in ad libitum fed **(A)** VTA LepR-Gq (n=6) and **(B)** SN LepR-Gq (n=6) or **(C)** food restricted VTA LepR-Gq mice (n=6). **(D)** CNO decreased locomotion in food restricted SN LepR-Gq mice (n=6). Mean ± SEM. *p<0.05.

Locomotor activity was assessed by automatically tracking horizontal movement of mice for 1h. CNO injections did not affect locomotor activity in ad libitum fed VTA LepR-Gq mice (**Figure 8A**) or SN LepR-Gq mice (**Figure 8B**). Activating VTA LepR-Gq neurons in food restricted mice did not modulate locomotion (**Figure 8C**), but activation of SN LepR-Gq neurons decreased locomotor activity (interaction effect of Time x Injection *F*(1.184,5.920)=9.380, p=0.020, **Figure 8D**). Further analyses revealed that CNO decreased locomotion after 40 and 60min in SN LepR-Gq mice (40min, p=0.031; 60min, p=0.025).

### Chemogenetically Activating VTA LepR or SN LepR Did Not Affect Anxiety-Like Behavior

**Figure 9:**
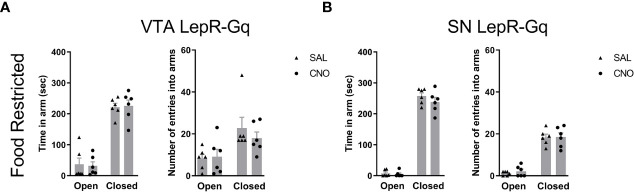
Anxiety-like behavior upon chemogenetic activation of VTA LepR or SN LepR neurons. Anxiety-like behavior in the EPM was not affected by CNO (1.0mg/kg) VTA LepR-Gq (n=6) **(A)** and SN LepR-Gq (n=6) **(B)** injected mice.

Finally, because previous studies showed that VTA LepR modulated anxiety (20, 28), we aimed to verify this in our experiments and determined effects of activating VTA LepR or SN LepR on anxiety-like behavior. To test for anxiety-like behavior, we examined behavior in the elevated plus maze (EPM) for 5min. This test is based on the natural aversion of mice to brightly lit, open and elevated spaces and we therefore report time spent in the avoided part of the EPM, i.e. the brightly lit and elevated open arms. Chemogenetic activation of VTA LepR or SN LepR in food restricted mice did not affect the amount of time spent in the open arm of the EPM (**Figure 9**). Although we increased the time between 2 test days to 3 weeks, anxiety-like behavior may be reduced with repeated exposure to the tasks, so we also analyzed individual test days. However, we did not observe reduced anxiety-like behavior on the second test day. When analyzing the data from the first test day only (comparing between subjects effects), we found no effect of CNO either (data not shown).

## Discussion

Leptin suppresses feeding, locomotion, anxiety and motivation to obtain food (15, 20, 28, 35). As the midbrain dopamine system has been implicated in these behaviors as well, we here addressed whether selective chemogenetic activation of leptin receptor expressing midbrain neurons modulated feeding, locomotion, anxiety and motivation. As we confirmed in this study, leptin receptors are expressed on a subset of VTA and SN neurons. Chemogenetic activation of VTA LepR neurons modulated motivation and feeding, whereas activation of SN LepR neurons modulated locomotion. Activation of neither population altered anxiety-like behavior.

Chemogenetic activation of VTA LepR neurons decreased motivational behavior for food reward. The decrease in motivation was a surprising result as most VTA LepR and SN LepR neurons are DAergic. Increased midbrain DA activity is associated with enhanced motivation (12–14) and chemogenetic activation of VTA DA neurons increases motivation (33). Contrary to our hypotheses, our findings support VTA LepR. A previous study found no effect of leptin infusion into the VTA on motivation in a operant responding task of ad libitum fed rats (21). However the lack of an effect in the study by Davis et al. may be due to the facts that these animals were ad libitum fed. Ad libitum animals have more fat than food restricted animals. Since leptin levels correlate with body fat (3, 5), endogenous leptin levels have been found elevated in ad libitum fed compared to food-restricted animals (5). Thus in ad libitum fed rats endogenous leptin levels are high and may have masked the effect of intra-VTA leptin on motivation. As we found a decrease in motivation by chemogenetically activating LepR-VTA neurons, VTA DA-LepR neurons, most of which project to the amygdala (18), not likely play a role in motivation for food reward. VTA GABA neurons are known to provide local inhibition of DA neurons and modulate reward behavior (36). One interpretation of our results is, therefore, that decreased motivation is the result of chemogenetic activation of VTA GABA-LepR neurons that reduce VTA DA activity. Indeed, it was recently found using optogenetic-assisted circuit mapping that VTA GABA neurons expressing the leptin receptor are activated by leptin and directly inhibit VTA DA neurons (37). Similarly, inhibitory input from the lateral hypothalamus to the VTA was shown to inhibit GABA neurons and increase dopamine release in the nucleus accumbens (38, 39). Thus by activating VTA GABA neurons as we have done, one would expect a decrease in motivational behavior in line with our results.

Our data further show that activating LepR neurons in the VTA decreased feeding in food restricted mice. Although the decrease in our study is minimal, this finding is in line with previous studies that reported decreased feeding upon intra-VTA leptin injections (15, 24, 40–42). Interestingly, previous studies reports suggest that non-DA neurons of the VTA mediate leptin effects on feeding: strategies targeting only DA LepR neurons, i.e. LepR deletion in DAT neurons (28) or STAT3 deletion in DAT neurons (27), did not affect feeding and mice with STAT3 deletion in DAT neurons remained responsive to leptin’s anorexic effect upon intra-VTA leptin infusions (27). Evenmore, a recent study showed that inhibition of VTA-GABA neurons decreased regular chow intake (43). Our data therefore support the idea that VTA GABA-LepR neurons, like VTA-GABA neurons, modulate feeding, as the decrease in feeding in our experiments is most likely mediated by the activation of these inhibitory neurons.

Given that most SN LepR neurons are DA neurons and chemogenetic activation of SN DA neurons increased locomotion (33), it was unexpected to find that activating SN LepR neurons decreased locomotion. An explanation for this could lie in the fact that only 15% of SN dopaminergic contain LepR and that 10% of SN-LepR neurons are also non-dopaminergic. This suggests that activation of this relatively low amount of DA neurons in the SN is not sufficient to increase locomotion, and also that the few inhibitory SN LepR neurons reduce locomotion. Furthermore, we found no effect of VTA LepR activation on locomotion. However, increased locomotion observed after LepR knockdown in VTA neurons (15) or loss of STAT3 in VTA DA neurons (27) suggest that VTA DA neurons that express LepR modulate locomotion. Similar to the explanation for SN neurons, perhaps activating a low amount of VTA DA neurons was similarly not sufficient to modulate locomotion.

We did not observe changes in anxiety-like behavior upon chemogenetic activation of VTA LepR. However, previous studies report that leptin reduces anxiety by inhibition of VTA DA-LepR neurons that project to the extended central amygdala (18, 20, 28). Furthermore, increased anxiety was seen after deletion of LepR in DA neurons and this was accompanied by increased burst firing of VTA DA neurons (28). Other studies have also suggested that burst firing in VTA DA neurons results in anxiogenic behaviour (44, 45). Together, these results suggest that chemogenetic activation of VTA LepR neurons did not activate VTA DA neurons to the extent of burst firing that is capable of increasing anxiety.

Interestingly, behavioral effects of chemogenetic activation of VTA LepR or SN LepR were only observed in food restricted mice, which have lowered leptin levels (35). As such, we assume that the neurons responsible for changing behavior show decreased activity when leptin levels are low. Since neuronal stimulation resulted in the reduction of the behavior tested, the observed effects were likely driven by the activation of inhibitory non-DA neurons in the VTA or SN, such as GABA neurons. Together, this suggests that GABA neurons mediated the observed reductions in behavior and that GABA neurons are less active with lower leptin levels. Therefore, we propose that leptin stimulates activity of these LepR neurons to contribute to the suppression of motivation, feeding and locomotion. Future studies will be needed to verify this.

There are limitations to the interpretation of the results. The number of mice used in the studies was limited with groups sizes of 6 mice and we performed most experiments with a single dose of CNO. As control for off-target effects of CNO we used mice that expressed a fluorescent protein (channelrhodopsin) not activated by CNO. We cannot exclude that larger group sizes, more doses of CNO or the use of other DREADD receptor ligands for which no off-target effects have been reported would result in unmasking roles of midbrain leptin receptor neurons in the assays we used. Therefore we hope that this study inspires others to replicate and extend our findings.

To conclude, our results show that activating SN LepR neurons decreased locomotion and activating VTA LepR neurons decreased feeding and motivation for food reward. Although both SN LepR and VTA LepR neurons are predominantly DA neurons, which are inhibited by leptin, the effects described here support involvement of inhibitory non-DA neurons in feeding, locomotion and food reward. This is an open question that needs to be addressed to more fully understand the mechanism underlying leptin’s effect on motivation for food reward.

## Data Availability Statement

The original contributions presented in the study are included in the article/**Supplementary Material**. Further inquiries can be directed to the corresponding author.

## Ethics Statement

The animal study was reviewed and approved by Animal Welfare Body Utrecht Nieuw Gildestein, room 1.81 Bolognalaan 50 3584 CJ Utrecht Phone: (030) 253 15 69 E-mail: info@ivd-utrecht.nl Website: www.ivd-utrecht.nl.

## Author Contributions

AO performed histology and confocal imaging. VV performed behavioral experiments, histology, and data analysis. ML-B performed stereotaxic virus/implant surgeries. LS and AR contributed to behavioral experiments. RA designed the experiments and wrote the manuscript with inputs from all authors. All authors contributed to the article and approved the submitted version.

## Funding

This research was supported by the European Union Seventh Framework Programme (grant agreement number 607310; Nudge-it), by the Netherlands Organisation for Scientific Research (grant number ALWOP.137) and by the Swedish Research Council (2018-02588).

## Conflict of Interest

The authors declare that the research was conducted in the absence of any commercial or financial relationships that could be construed as a potential conflict of interest.
