# Supplementary material for: Leptin Receptor Expressing Neurons in the Substantia Nigra Regulate Locomotion, and in The Ventral Tegmental Area Motivation and Feeding
Source: Front Endocrinol (Lausanne). 2021 Jul 1;12:680494. doi: 10.3389/fendo.2021.680494 (PMC8281287; doi:10.3389/fendo.2021.680494)

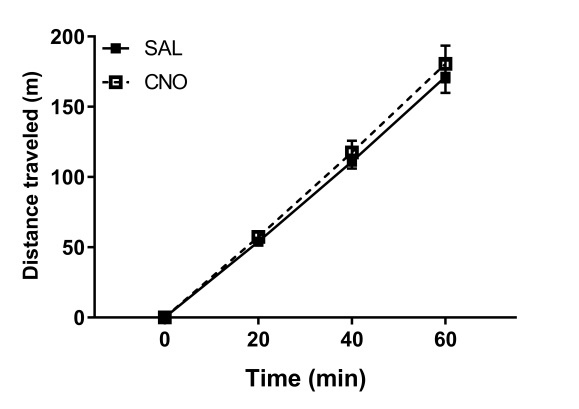

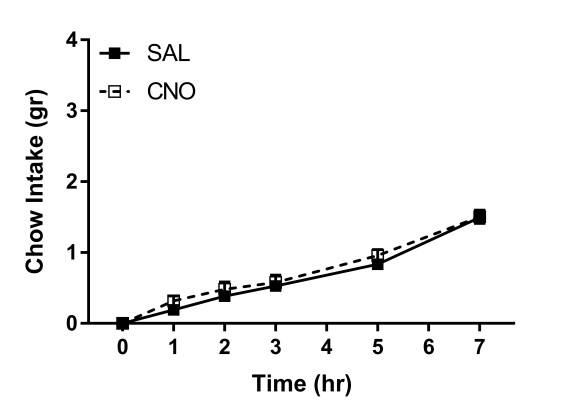

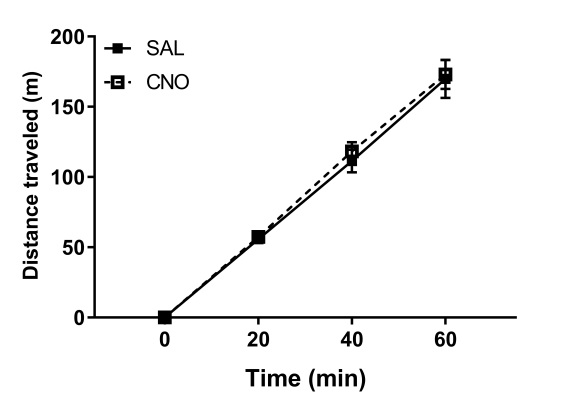

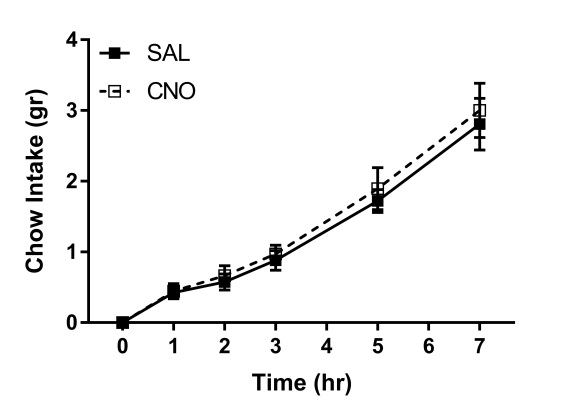

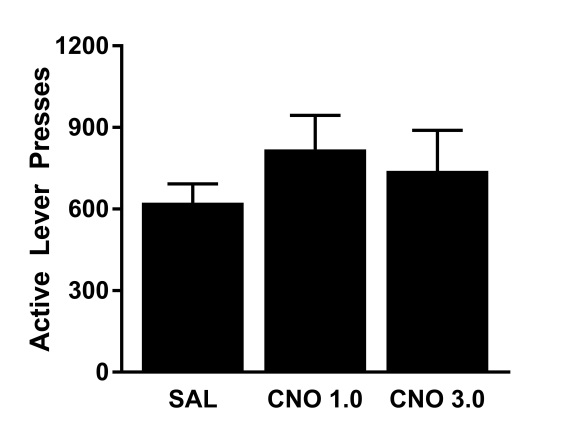

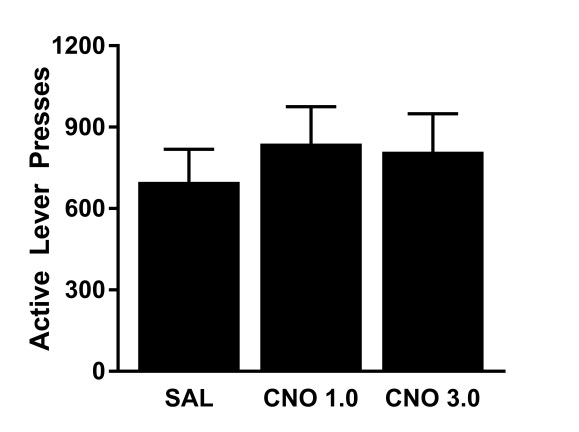


Ad Libitum

Food Restricted

**Supplemental figure. Behavioural effects of CNO in control mice.** In control LepR-cre mice injected with AAV-Ef1a-DIO-hChR2-eYFP (n=6) CNO injections had no effect on (A, B) active lever presses, (C, D) locomotion, (E, F) chow intake, (G, H) locomotion or (I) anxiety-like behaviour. Mean ± SEM.


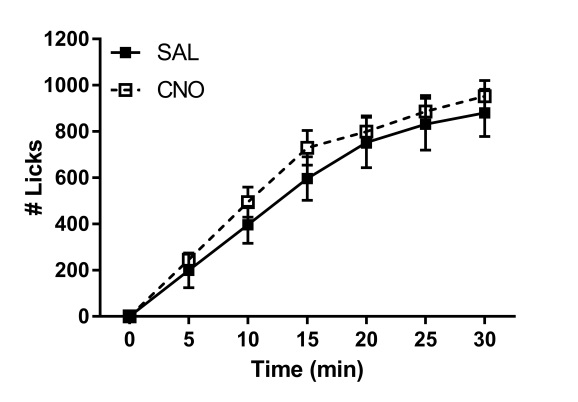

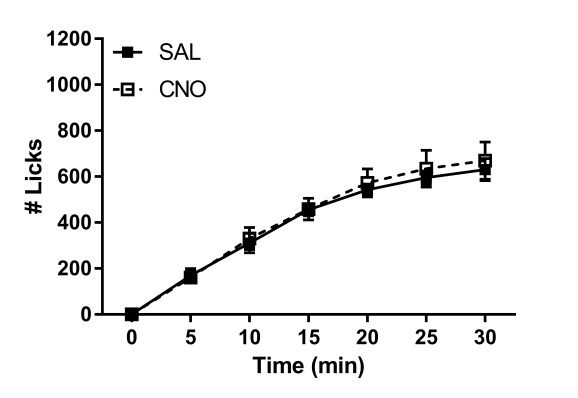


A

B

C

D

E

F

G

H

I


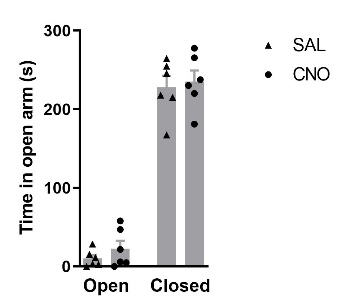

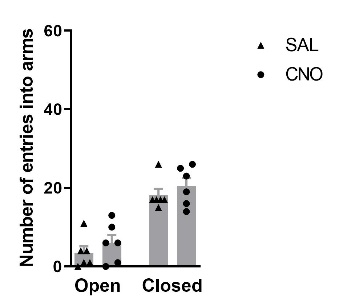

Supplement: Supplementary file 1 [file DataSheet_1.docx]
